# Influence of eicosapentaenoic acid supplementation on lean body mass in cancer cachexia

**DOI:** 10.1038/bjc.2011.391

**Published:** 2011-10-04

**Authors:** R A Murphy, E Yeung, V C Mazurak, M Mourtzakis

**Affiliations:** 1 Division of Human Nutrition, Department of Agricultural, Food and Nutritional Science, University of Alberta, 4-126 Li Ka Shing Center, Edmonton, AB T6G 2E1, Canada; 2 Department of Kinesiology, Faculty of Applied Health Sciences, University of Waterloo, 200 University Avenue W, Waterloo, ON N2L 3G1, Canada

**Keywords:** n-3 fatty acids, sarcopenia, fish oil, muscle loss

## Abstract

Cancer cachexia is characterised by a progressive loss of muscle, resulting in functional impairment and shorter survival. Eicosapentaenoic acid, an n-3 polyunsaturated fatty acid found in fish, has been studied for its role as an anti-cachexia therapy. Initial results of eicosapentaenoic supplementation in advanced cancer were promising with improvements in lean body mass (LBM), appetite and quality of life. However, subsequent larger phase III clinical trials reported minimal benefits of supplementation. Recently, several studies have used different study designs, which may provide insight on the effectiveness of eicosapentaenoic in cancer cachexia and also on potential sources of divergent results in previous trials. This review examines the potential benefit of eicosapentaenoic supplementation on LBM and discusses limitations with current studies to identify methods which may aid in progressing the research of future clinical trials.

Cachexia is a complex metabolic syndrome characterised by unintentional weight loss and depletion of skeletal muscle, with or without loss of adipose tissue (Evans *et al*, 2008). Cachexia is prevalent in chronic or end-stage cancers, and nearly half of all cancer patients experience some degree of weight loss (Dewys *et al*, 1980). Clinical manifestations of cachexia include: reduced strength, fatigue, impaired function and poor quality of life (Dodson *et al*, 2011).

The search for effective anti-cachexia therapy has been challenging and is an area of intense research. One commonly studied intervention is fish oil, consisting of n-3 polyunsaturated fatty acids, eicosapentaenoic acid (EPA) and docosahexaenoic acid (DHA). Through the time-course of studies that have examined the potential benefits of supplementation with EPA and DHA or EPA alone in cancer cachexia, we have identified divergent results of EPA on lean body mass (LBM). LBM primarily consists of skeletal muscle, with the remainder comprised of metabolic tissues (i.e., kidney and liver), and intracellular and extracellular water. LBM is commonly used as a surrogate measure of skeletal muscle in clinical trials in the absence of methods such as computed tomography (CT) and magnetic imaging resonance, which are capable of distinguishing skeletal muscle. In this review, we focus on the effect of EPA on LBM or skeletal muscle when assessed directly, as depletion of skeletal muscle is the defining characteristic of cachexia (Evans *et al*, 2008; Fearon *et al*, 2011). As well, there is an association between loss of muscle and proximity to death (Lieffers *et al*, 2009), and changes in muscle are reflective of changes in strength and physical function (Frontera *et al*, 1988). Hence, examining the effectiveness of intervention with EPA in attenuating deleterious losses of LBM is essential, and identifying gaps in the literature is fundamental for guiding future research trials.

Early investigational trials of supplementation with either fish oil or EPA alone in weight-losing cancer patients yielded promising results. Benefits to patients included preservation of LBM, increased physical activity, improved appetite and weight gain (Gogos *et al*, 1998; Barber *et al*, 1999; Wigmore *et al*, 2000; Moses *et al*, 2004). However, these studies were generally small, non-randomised and uncontrolled. Subsequent phase III, large, randomised clinical trials failed to show the benefit of EPA over placebo on LBM (Fearon *et al*, 2003, 2006; Jatoi *et al*, 2004). A systematic review on the subject (Dewey *et al*, 2007) further dampened enthusiasm for EPA as an anti-cachexia therapy, concluding that there is insufficient evidence that EPA provides a benefit over placebo on cancer cachexia and related symptoms. However, in the last 2 years, several studies have again pointed towards potential benefits of EPA for attenuating LBM loss, as well as maintenance or gain of LBM.

The purpose of this short review is to discuss the potential causes for the discrepancy in results between previous EPA supplementation studies, and examine these causes in relation with more recent clinical studies. Understanding the limitations of current studies and discrepancies throughout the literature is fundamental for proceeding with future studies.

Although the focus of this review is the effect of EPA on LBM, EPA may also independently improve function and physical activity (Moses *et al*, 2004). Although a full discussion on these outcomes is beyond the scope of this review, there is a relationship between muscle mass, strength and function (Frontera *et al*, 1988), and it is likely that improvements in LBM also reflect improvements in muscle function.

## Potential mechanisms of dietary EPA on LBM

**Figure 1:**
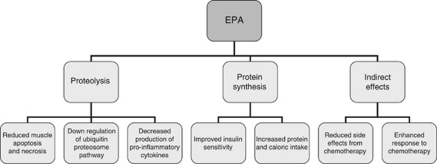
Studies suggest that EPA affects LBM via several diverse mechanisms including effects on proteolysis, protein synthesis, as well as indirect effects, which may all lead to attenuation, maintenance or gain in LBM.

To better understand the discrepancies that have been reported in the literature, the proposed mechanisms of action of dietary EPA on LBM will be discussed briefly. The aetiology of muscle loss in cancer is complex and is likely the net result of several tumour-derived and host-derived factors (reviewed in Tisdale, 2009). Likewise, EPA appears to act on several direct and indirect pathways, which contribute to muscle wasting and muscle anabolism (Figure 1).

EPA may support the anabolic potential of muscle through sensitising skeletal muscle to insulin. Insensitivity to insulin has been observed in patients with cancer cachexia (Dodesini *et al*, 2007) and may contribute to the development of cachexia. In tumour-bearing mice, insulin insensitivity preceded weight loss and administration of Rosiglitazone, a drug used in the treatment of type 2 diabetes, improved insulin sensitivity and attenuated skeletal muscle proteolysis (Asp *et al*, 2010). In experimental models of diabetes, EPA has been shown to improve glucose uptake and increase GLUT-4 expression in skeletal muscle. However, to our knowledge, this relationship has not been explored in cancer cachexia, and the precise points of EPA interaction within the glucose–insulin-signalling pathway in muscle remains unclear.

Conversely, EPA has been shown to inhibit several catabolic stimuli that promote muscle degradation during the cachectic process. The acute-phase protein response may contribute to muscle wasting, as it is modulated in part by pro-inflammatory cytokines: IL-1, IL-6, TNF-*α*, IFN-*γ* (McNamara *et al*, 1992). Supplementation with EPA may limit muscle degradation by downregulating the acute-phase response. In weight-losing cancer patients, EPA has been shown to reduce serum concentrations of C-reactive protein, an acute-phase protein, and suppress IL-6 production by peripheral blood mononuclear cells (Wigmore *et al*, 1997). The ubiquitin-proteasome proteolytic pathway is another key contributor to muscle breakdown in cancer-cachexia. EPA may decrease muscle breakdown by decreasing the expression of proteasome subunits, which are elevated in cancer cachexia (Tisdale, 2009). EPA may further decrease muscle breakdown via a protective role in the skeletal muscle differentiation. An *in vitro* study showed reduced necrosis and apoptosis of differentiating myotubes, with addition of EPA to the media (Magee *et al*, 2008). In the same model, addition of EPA completely abolished the TNF-α induced necrosis and apoptosis. The effects of EPA on catabolic stimuli are diverse, and further research is required to determine under what conditions these pathways are activated and how to optimise the inhibitory effect of EPA on muscle degradation.

Side effects from anti-neoplastic therapies may contribute to or exacerbate existing anorexia, leading to a negative energy balance and muscle wasting. In an animal model of colorectal cancer, providing EPA and DHA reduced the side effects from chemotherapy, and limited weight loss and anorexia (Xue *et al*, 2007). EPA and DHA have also been reported to enhance tumour response to chemotherapy, thereby reducing the disease burden (Bougnoux *et al*, 2010; Murphy *et al*, 2011b). This may indirectly provide anabolic stimuli as improvements in functional activity, dyspnoea, fatigue and physical function, and have been observed in patients receiving chemotherapy (reviewed in Klastersky and Paesmans, 2001). Protein and caloric intake have been reported to be improved with EPA, which may also influence muscle mass (Fearon *et al*, 2003). Thus, there are various experimental and observational studies in animals and humans that demonstrate positive results in the use of EPA to attenuate symptoms of cachexia.

## Clinical studies: the effect of EPA on LBM

The beneficial effects of EPA supplementation on LBM that were reported in early studies of EPA supplementation (Barber *et al*, 1999; Wigmore *et al*, 2000) are a stark contrast to subsequent phase III trials, which showed no benefit of EPA over placebo on LBM (Fearon *et al*, 2003, 2006; Jatoi *et al*, 2004). Recently, four clinical trials (Ryan *et al*, 2009; van der Meij *et al*, 2010; Murphy *et al*, 2011a; Weed *et al*, 2011) reported beneficial effects of EPA supplementation, including gain, maintenance or milder loss of LBM than corresponding control groups (summarised in Table 1). This review focuses on these studies because they feature study designs, which are distinct from previous trials. Differences in study designs may help to explain discrepant results across EPA studies in the last decade. Specifically, study compliance, measures of phospholipid (PL) EPA concentrations, assessment of LBM and timing of intervention will be examined in relation to previous trials.

### Compliance

Compliance to the study intervention is a ubiquitous challenge. Several studies have reported poor compliance to EPA supplementation, with intakes well below the prescribed amounts (Bruera *et al*, 2003). Contamination between treatment arms has also been reported: increased PL EPA with placebo and no increase in PL EPA with supplementation (Fearon *et al*, 2003, 2006), demonstrating the difficulties in conducting these studies, as patients who understand the potential benefits of EPA may be driven to take EPA supplementation on their own initiative. Several recent studies used study designs that may mitigate poor compliance and contamination between treatment arms. Murphy *et al* (2011a) conducted two separate contemporaneous trials; one, which was a standard-of-care description of changes in body composition, and another one, which offered patients the choice between two formats of EPA supplementation (capsules or liquid). They reported no instances of self-supplementation in the standard-of-care study (assessed by plasma PL EPA concentration) and over 95% compliance to the EPA supplement. Ryan *et al* (2009) used EPA-enriched parenteral nutrition, and patients from the study by Weed *et al* (2011) received the supplement via tube feed.

### Heterogeneity of plasma PL EPA following supplementation

The majority of n-3 fatty acids in blood are contained in plasma PL. Accordingly, the concentration of EPA in plasma PL is commonly used as a measure of compliance to EPA supplementation (Bruera *et al*, 2003; Fearon *et al*, 2006). Previous studies have shown mean increases in PL EPA post-supplementation (Barber *et al*, 1999; Wigmore *et al*, 2000). However, the extent of these increases varied, and one study reported little to no change in PL EPA in 25% of patients, despite reported compliance (Fearon *et al*, 2003). It was assumed that variation was due to misreporting of intake, but additional studies have also identified variation in PL n-3 fatty acids beyond, which could be explained by misreporting supplement intake (Bougnoux *et al*, 2010; Murphy *et al*, 2011a). The reason for lack of incorporation into cellular membranes is unclear, but may be related to proximity to death as PL n-3 fatty acids have been reported to decrease approaching time of death in advanced cancer patients (Murphy *et al*, 2010). As EPA must be digested, absorbed and subsequently incorporated into cells and tissues to exert physiological functions, it is possible that unless low incorporation is controlled for, differential incorporation of EPA may dilute the effect of EPA on LBM. In the study by Murphy *et al* (2011a), EPA supplementation resulted in overall maintenance of muscle. However, a portion of patients lost muscle, despite compliance to the supplement. Using linear regression, the authors showed that over half of the variability in muscle change was explained by plasma EPA concentrations; the patients who lost muscle were the patients who had minimal changes in PL EPA concentration. This is a simple approach that may help to identify the true effect of EPA on LBM.

### Assessment of change in LBM lacks specificity

There are several important methodological limitations to consider when choosing a body composition assessment tool. Bioelectrical impedance (BIA) is appealing, because it is portable and cost effective. However, BIA and other methods of LBM assessment such as skin folds cannot distinguish between skeletal muscle and other lean tissues. BIA also relies on predictive equations to generate an estimate of lean tissue, but equations that are representative of cancer populations are limited. In a comparison of LBM assessment using BIA and dual-energy X-ray absorptiometry (DXA), discrepancies ranged from −9.3 to +7.3 kg, with overestimation likely in patients with low LBM, and underestimation likely in patients with high LBM (Mourtzakis *et al*, 2008). DXA, but not BIA, detected small but clinically significant changes in LBM. Thus, changes in LBM may not have been detected in previous studies of EPA supplementation, which utilised BIA.

In advanced cancer, mean gain in liver and spleen mass of nearly 1 kg has been observed (Lieffers *et al*, 2009), which may disguise changes in skeletal muscle if LBM rather than skeletal muscle is measured. Thus, specific discrimination of skeletal muscle is important for distinguishing between gain in LBM that is attributable to visceral organs and gain that is attributable to skeletal muscle. CT imaging can discriminate muscle, adipose tissues depots, bone and other organs with a precision error of ∼2% (Mourtzakis *et al*, 2008). CT imaging is routinely used in oncology settings for disease staging and follow-up purposes, and can be opportunistically used to specifically quantify skeletal muscle. Although CT images are clinically accessible, this is a relatively new area of research in cancer, and to date, only one study has utilised CT imaging to assess the effect of EPA supplementation on skeletal muscle (Murphy *et al*, 2011a).

### Timing of intervention with EPA

An international panel of experts on cachexia recently developed a classification system, which recognises that cachexia occurs across a continuum, varying in severity and stage: (1) pre-cachexia: early clinical or metabolic signs of cachexia, low-grade weight loss, which may progress to cachexia, (2) cachexia: weight loss >5% in the last 6 months or a combination of >2% weight loss with low muscle or low BMI, and (3) refractory cachexia: occurs close to death due to rapidly progressing disease, which is unresponsive to anti-cancer therapy (Fearon *et al*, 2011). In refractory cachexia, accelerated loss of tissues amounting to deficits of more than 4 kg of both muscle and adipose tissue have been reported (Lieffers *et al*, 2009; Murphy *et al*, 2010). It is unlikely that these deficits can be overcome, and it has been suggested that anti-cachexia therapy should be diverted away from the end of life when the burden of intervention likely outweighs any benefits (Fearon *et al*, 2011). Thus, it is critical that research looks to interventions that can be initiated during the pre-cachexia stage, with the aim of preventing deleterious losses of muscle.

Traditionally, cachexia has been viewed as an end-of-life condition, but cachexia and related muscle loss may occur early in the disease trajectory. In the study by Murphy *et al* (2011a), which included newly diagnosed early and advanced-stage lung cancer, 46% of patients had severe muscle depletion at the start of the study and it was unchanged following EPA supplementation, but increased to 63% in the control group. This is a clear illustration of the importance of early intervention. Further, the studies in Table 1 were conducted in patients newly diagnosed with lung cancer or patients with cancers of the lung, head and neck or oesophagus, who were receiving curative treatment and/or were hospitalised. In these patient populations, there is a reasonable expectation that patients will lose weight and muscle during the disease trajectory, even if they have not done so at the moment of first referral and thus, may benefit from early intervention. As such, with the exception of the study by Weed *et al* (2011), none of the studies in Table 1 had weight loss as an inclusion criterion. These patients had generally mild or insignificant weight loss, and may have been in the pre-cachexia stage. This approach also enabled accrual of patients with better survival prospects, who are more likely to benefit from cachexia therapy (Fearon *et al*, 2011). The patient populations in Table 1 also consisted of cancer types (oesophageal, lung, and head and neck) that have a greater expected median survival and generally less intense wasting than patients with pancreatic cancer, who comprised the study population in the majority of the earlier studies (Barber *et al*, 1999; Wigmore *et al*, 2000).

Refractory cachexia may have been present in the patient populations of previous EPA trials. Despite general inclusion criteria of life expectancy >3 months, previous EPA trials have been plagued by significant patient morbidity and loss of patients due to disease progression (Wigmore *et al*, 2000; Bruera *et al*, 2003; Fearon *et al*, 2003). Short median survival ranging from 6 months to less than 3 months from onset of supplementation has also been reported in numerous studies (Bruera *et al*, 2003; Fearon *et al*, 2003, 2006). Recent or active treatment with anti-neoplastic therapy is a common exclusion criterion (Fearon *et al*, 2006); however, this has the pitfall of including patients with progressive or refractory disease. Conversely, many studies do not exclude patients with intense wasting, and losses of up to 56% of body weight have been reported (Fearon *et al*, 2006). These are a different class of patients in that they are typically not eligible for anti-neoplastic therapy due to poor performance status or concurrent progressive disease and are likely close to death. Therefore, EPA supplementation may be expected to be more effective when implemented early in the disease trajectory versus later.

## Conclusions and recommendations

In summary, it seems likely that the encouraging results from Ryan *et al* (2009), van der Meij *et al* (2010), Weed *et al* (2011) and Murphy *et al* (2011a) are related to features of their study design. On the basis of these studies, we suggest several points of consideration for the design of future trials of EPA. First, offering patients a choice of supplementation format (capsules or liquid), or use of EPA-enriched parenteral or enteral nutrition may be an effective means to improve study compliance. Studies should also consider stratifying outcomes according to the PL EPA to account for differential incorporation of EPA into PL. CT image analysis should be incorporated as a study outcome measure whenever possible, as it is expedient in an oncology setting and can precisely quantify skeletal muscle. Finally, EPA supplementation may be more effective if provided earlier rather than later, when muscle loss is accelerated. Early intervention allows for accrual of patients who likely have better survival prospects, and for whom maintenance or muscle gain is more likely. We hope the results of the recent studies of fish oil intervention in cancer patients, reviewed here, will help to inform the design of future studies and encourage continual investigation on the use of EPA as a therapy to prevent muscle loss.

## Figures and Tables

**Table 1:** Summary of recent clinical trials on the effect of eicosapentaenoic acid on lean body mass

**Author**	**Design**	**Population**	**Intervention**	**LBM assessment**	**Results**
Murphy *et al* (2011a)	Open-label, single arm with contemporaneous control group	31 Patients with mixed-stage non-small cell lung cancer receiving chemotherapy. 24 in control (C) and 16 in intervention (I) group.	I: Four 1-g capsules per day (2.5 g EPA + DHA) or 7.5 ml syrup per day (2.5 g EPA + DHA) for the duration of chemotherapy ∼10 weeks. C: no intervention. Mean intake: 2.4 g per day.	Computed tomography image analysis.	I: Overall maintenance of weight and skeletal muscle, 69% gained or maintained muscle. Muscle gain in patients with the largest increase in plasma PL EPA was related to the rate of muscle change. C: weight loss (−2.3 kg) and muscle loss (−1 kg), 29% gained or maintained muscle.
Weed *et al* (2011)	Open-label, single arm	31 Weight-losing patients with head and neck cancer undergoing curative intent resection.	Two cans enriched-ONS per day (2.2 g EPA). Mean intake: 1.8 cans per day, pre-op and during hospitalisation ∼5 weeks.	BIA	Significant increase in LBM (+3.2 kg) and significant decrease in fat mass (−3.2 kg).
van der Meij *et al* (2010)	Randomised controlled, blinded	33 Patients with stage III non-small cell lung cancer receiving adjuvant chemoradiation. 19 in intervention (I) and 14 in control (C) group.	I: Two cans of enriched-ONS per day (2 g EPA + 0.9 g DHA) for 5 weeks. Mean intake: 1.1 can per day. C: control ONS. Mean intake: 1.0 can per day for 5 weeks.	BIA, MUAC	I: Weight maintenance, increased MUAC, decreased serum IL-6 and CRP in patients with ⩾1.5% increase in plasma PL EPA. Greater decrease of REE in I *vs* C. Milder decrease of FFM in I *vs* C.
Ryan *et al* (2009)	Randomised, controlled, blinded	53 Patients with localised oesophageal cancer receiving surgery, or surgery, chemotherapy and radiation. 28 in intervention (I) and 25 in control (C) group.	I: EPA-enriched enteral feed (2.2 g EPA per day) for 26 days. C: iso-caloric, iso-nitrogenous standard feed. All patients tolerated enteral feeding for 26 days.	BIA	I: Maintenance of LBM. 8% muscle loss >5% of body weight. C: 1.9 kg loss of LBM. 39% muscle loss >5% of body weight. No difference in CRP, albumin or IL-6 between groups.

Abbreviations: BIA=bioelectrical impedance analysis; CRP=C-reactive protein; DHA=docosahexaenoic acid; EPA=eicosapentaenoic acid; LBM=lean body mass; MUAC=mid upper arm circumference; ONS=oral nutritional supplement; PL=phospholipid; REE=resting energy expenditure.
